# Two variants on T2DM susceptible gene HHEX are associated with CRC risk in a Chinese population

**DOI:** 10.18632/oncotarget.8865

**Published:** 2016-04-20

**Authors:** Rui Sun, Jian-Ping Liu, Chang Gao, Ying-Ying Xiong, Min Li, Ya-Ping Wang, Yan-Wei Su, Mei Lin, An-Li Jiang, Ling-Fan Xiong, Yan Xie, Jue-Ping Feng

**Affiliations:** ^1^ Department of Oncology, PuAi Hospital of Tongji Medical College, Huazhong University of Science and Technology, Wuhan, China; ^2^ Department of Clinical Laboratory, PuAi Hospital of Tongji Medical College, Huazhong University of Science and Technology, Wuhan, China; ^3^ Department of Endocrinology, Wuhan PuAi Hospital of Tongji Medical College, Huazhong University of Science and Technology, Wuhan, China; ^4^ Department of Medicine, Washington University School of Medicine, St. Louis, Missouri, United States of America

**Keywords:** type 2 diabetes mellitus, HHEX, colorectal cancer, genetic variants

## Abstract

Increasing amounts of evidence has demonstrated that T2DM (Type 2 Diabetes Mellitus) patients have increased susceptibility to CRC (colorectal cancer). As HHEX is a recognized susceptibility gene in T2DM, this work was focused on two SNPs in HHEX, rs1111875 and rs7923837, to study their association with CRC. T2DM patients without CRC (T2DM-only, n=300), T2DM with CRC (T2DM/CRC, n=135), cancer-free controls (Control, n=570), and CRC without T2DM (CRC-only, n=642) cases were enrolled. DNA samples were extracted from the peripheral blood leukocytes of the patients and sequenced by direct sequencing. The χ^2^ test was used to compare categorical data. We found that in T2DM patients, rs1111875 but not the rs7923837 in HHEX gene was associated with the occurrence of CRC (p= 0.006). for rs1111875, TC/CC patients had an increased risk of CRC (p=0.019, OR=1.592, 95%CI=1.046-2.423). Moreover, our results also indicated that the two variants of HEEX gene could be risk factors for CRC in general population, independent on T2DM (p< 0.001 for rs1111875, p=0.001 for rs7923837). For rs1111875, increased risk of CRC was observed in TC or TC/CC than CC individuals (p<0.001, OR= 1.780, 95%CI= 1.385-2.287; p<0.001, OR= 1.695, 95%CI= 1.335-2.152). For rs7923837, increased CRC risk was observed in AG, GG, and AG/GG than AA individuals (p< 0.001, OR= 1.520, 95%CI= 1.200-1.924; p=0.036, OR= 1.739, 95%CI= 0.989-3.058; p< 0.001, OR= 1.540, 95%CI= 1.225-1.936). This finding highlights the potentially functional alteration with HHEX rs1111875 and rs7923837 polymorphisms may increase CRC susceptibility. Risk effects and the functional impact of these polymorphisms need further validation.

## INTRODUCTION

Accumulated evidence has demonstrated that diabetes is a risk factor of various types of cancers such as colorectum (RR=1.3) [1], breast (RR=1.2) [2], endometrium (RR=2.1) [3], bladder (RR=1.35) [4], liver (RR=2.5) [5], and pancreas (RR=1.94) [6] cancer. Cancer patients with DM have a poorer prognosis than those without DM [7–9]. Type 2 diabetes mellitus (T2DM) have threaten more than 200 million individuals worldwide and its prevalence continues to increasing in many countries including China. Although the precise mechanisms underlying the development and progression of T2DM have not been elucidated, hereditary factors have been known to contribute to the T2DM. The first-degree relatives of T2DM patients have ~3.5 times the risk of T2DM compared to individuals in the general population [10, 11]. Besides obesity, high red meat consumption, cigarette smoking and alcohol abuse [12–15], diabetes was involved to hold responsible for the increased cancer incidence and worse prognosis of CRC [16–19]. Nevertheless whether hereditary factors of T2DM contribute to the risk of colorectal cancer is still unknown. It is also unclear whether those factors worsen the prognosis of T2DM.

The gene encoding hematopoietically expressed homeobox (HHEX) has been identified to be related to T2DM [20–25]. HHEX gene encodes a transcription factor involved in Wnt signal pathway fundamental for cell growth and differentiation [26–28]. Aberrant activation of Wnt/β-catenin signaling promotes the occurrence of colorectal cancer. Deregulation in Wnt/β-catenin-Tcf/Lef axis [29–34] is responsible for most CRCs [35–37]. Recently, converging interest in unraveling HHEX on the development of hyperglycemia has triggered hundreds of association studies. Polymorphisms of rs1111875 T>C and rs7923837 A>G in HHEX gene have been identified and evaluated. Moreover significant association of rs1111875 or/and rs7923837 with T2DM was observed in many studies [38–42].

Although the association between HHEX polymorphisms and T2DM has been well studied, the association of HHEX SNPs with CRC remains unclear. Therefore, the present study focuses on the association between HHEX polymorphisms and CRC risks in Chinese patients.

## RESULTS

### CRC risk was higher in T2DM patients than non-T2DM control

T2DM has been identified to be associated with increased risk for CRC [44, 45]. Similar to the previous reported research, we found that the CRC risk in T2DM patients was about 1.3 times higher than that in non-DM control (p=0.00282, OR=1.3097, 95%CI =1.097-1.564), Table 1.

**Table 1:** The increased CRC occurrence of T2DM patients

	CRC	non-CRC	p	OR CI (95%)
Non-T2DM	1053	94428	0.00282	1.3097 (1.097-1.564)
T2DM	140	9586		

### In T2DM patients, rs1111875 variants of HHEX were associated with the occurrence of CRC

Many studies have proved that T2DM was an independent risk factor of CRC [44–48], but the association between HHEX variants and the CRC were rarely studied. In this study, rs1111875 and rs7923837 variants of HHEX were analyzed (Table 2). Both passed the Hardy-Weinberg equilibrium exact test (P > 0.05) in selected samples. MAFs were similar to data of Han Chinese in Beijing (CHB) and populations from HapMap (http://hapmap.ncbi.nlm.nih.gov/). The detailed genotype and allele distributions of the rs1111875 and rs7923837 in the T2DM-only and T2DM/CRC patients are presented in Table 3.

**Table 2:** The characteristics of the rs1111875 and rs7923837 in HHEX gene

Polymorphisms	Alleles	MAF	HWE *
rs1111875	T>C	0.288	0.728
rs7923837	A>G	0.228	0.256

* Goodness-of-fit chi-square test was used to assess Hardy–Weinberg equilibrium (HWE) in controls.

**Table 3:** Association between the rs1111875, rs7923837 of HHEX and risk of CRC in T2DM patients

Polymorphisms	T2DM-only (300)		T2DM/CRC (135)		p	OR (95% CI)
rs1111875	n	%	n	%		
TT	162	58.1	60	46.5	**0.006**	1.00(reference)
TC	91	32.6	43	33.3	0.184 (*vs.* TT)	1.276 (0.798-2.037) (*vs.* TT)
CC	26	9.3	26	20.2	**0.0015 (*vs.* TT)**	2.700 (1.454-5.014) (*vs*. TT)
TC/CC	117	41.9	69	53.5	**0.019 (*vs.* TT)**	1.592 (1.046-2.423) (*vs.* TT)
T allele	415		146		**<0.001**	1.00(reference)
C allele	143		95			1.888 (1.370-2.602)
rs7923837						
AA	174	58.0	87	64.5	0.255	1.00(reference)
AG	111	37.0	45	33.3	0.340 (*vs.* AA)	1.233 (0.801-1.899) (*vs.* AA)
GG	15	5.0	3	2.2	0.229 (*vs.* AA) 0.414 (*vs.* AG)	2.500 (0.705-8.867) (*vs.* AA) 2.027 (0.560-7.342) (*vs.* AG)
AG/GG	126	42.0	48	35.5	0.204	1.312 (0.862-1.998)
A allele	459		219		0.129	1.00(reference)
G allele	141		51			1.319 (0.921-1.888)

Two-sided χ2-test for the distributions of either genotype or allele frequencies between the DM-only and DM/CRC crowd. Genotype-specific ORs were adjusted for age, gender, smoking status, drinking status in logistic regression model.

The frequency of the selected characteristics in 300 cases of T2DM-only and 135 T2DM/CRC patients was shown in Table S1. No significant differences regarding to age (p= 0.71), gender (p= 0.14), BMI (p= 0.06), hypertension (p= 0.26), smoking status (p= 0.73), were observed among the T2DM-only and T2DM/CRC. However, there were more drinkers in the T2DM/CRC than in the T2DM-only (p= 0.006). Since insulin and degree of glycemic control have been considered as risk factors in CRC development and progression [49], fasting serum glucose (FSG), insulin and glycated hemoglobin (HbA1c) were all involved in analysis. No significant differences regarding to these three factors between the two groups were observed. In the total 300 cases of T2DM-only and 135 T2DM/CRC patients, 21 cases of T2DM-only and 6 cases of T2DM/CRC patients were sequencing failure for rs1111875 because of the weak signal or no signal, the sequencing for rs7923837 was all successful. Significant difference in genotype of rs1111875 was observed between the T2DM-only and T2DM/CRC patients (p= 0.006). We found that the rs1111875 of HHEK was significantly associated with CRC risk. Compared to individuals with TT, those subjects with TC/CC had a significant increased CRC risk (p=0.019, OR=1.592, 95%CI=1.046-2.423), the subjects with CC had a significant increased CRC risk (p=0.0015, OR=2.700, 95%CI=1.454-5.014). Significant difference in allele frequency distribution was observed between T2DM-only and T2DM/CRC (p< 0.001 OR=1.888, 95%CI=1.370-2.602).

Another SNP (rs7923837) was not associated with CRC risk in T2DM patients (p=0.255). No significant difference in allele frequency distribution was observed between T2DM-only and T2DM/CRC (p=0.129). Taken together, these results indicate that rs1111875 but not rs7923837 in HHKE gene was associated with the occurrence of CRC in T2DM patients. Nonetheless, further questions of whether rs1111875 variant in HHKE gene enhance the occurrence of CRC was T2DM dependent or rs1111875 and rs7923837 could increase the CRC risk without the background of T2DM was soon taken into our consideration.

### HHEX variants rs1111875 and rs7923837 were CRC risk factors and independent on the occurrence of T2DM

Based on the results above, a research on health control cohort and CRC-only group was conducted.

The frequency distribution of the selected characteristics of 570 cases of control and 642 CRC-only patients were shown in Table S2. No significant differences regarding to age (p= 0.08), BMI (p= 0.29), gender (p= 0.09), hypertension (p= 0.09) and drinking status (p= 0.09) were observed among the control and CRC-only subjects. However, there were more smokers among the CRC-only than among the control (p= 0.04). No significant differences regarding to FSG, insulin and HbA1c was observed between the two groups. In the total 570 cases of control and 642 CRC patients, 33 cases of control and 81 cases of CRC-only patients were sequencing failure for rs1111875 because of the weak signal or no signal, the sequencing of rs7923837 was all successful. The detailed genotype and allele distributions of the rs1111875 and rs7923837 in the CRC-only patients and health controls were presented in Table 4.

**Table 4:** Association between the rs1111875, rs7923837 of HHEX and risk of CRC

Polymorphisms	Control(570)		CRC-only(642)		P*	Adjusted OR (95% CI)^#^
rs1111875	n	%	n	%		
TT	304	56.6	244	43.5	**<0.001**	1.00(reference)
TC	189	35.2	270	48.1	**<0.001 (vs TT)**	1.780 (1.385-2.287) (*vs.* TT)
CC	44	8.2	47	8.4	0.125 (*vs.* TT)	1.331 (0.853-2.075) (*vs.* TT)
TC/CC	233	43.4	317	56.5	**<0.001 (*vs*. TT)**	1.695 (1.335-2.152) (*vs.* TT)
T allele	797		758		**<0.001**	1.00(reference)
C allele	277		364			1.382 (1.148-1.663)
rs7923837						
AA	349	61.2	325	50.6	**0.001**	1.00(reference)
AG	200	35.1	283	44.1	**<0.001 (*vs.* AA)**	1.520 (1.200-1.924) (*vs.* AA)
GG	21	3.7	34	5.3	**0.036 (*vs.* AA)**	1.739 (0.989-3.058) (*vs.* AA)
AG/GG	221	38.8	317	49.4	**<0.001 (*vs.* AA)**	1.540 (1.225-1.936) (*vs.* AA)
A allele	898		959		**0.018**	1.00(reference)
G allele	242		325			1.258 (1.040-1.520)

* Two-sided χ2-test for the distributions of either genotype or allele frequencies between the control and case crowd. ^#^Genotype-specific ORs were adjusted for age, gender, smoking status, drinking status in logistic regression model.

Significant difference in genotype and allele distributions of both rs1111875 and rs7923837 were observed between the control and CRC-only (p< 0.001, p=0.001). For rs1111875, compared with individuals with TT, TC or TC/CC individuals had a significant increased CRC risk (p<0.001, OR= 1.780, 95%CI= 1.385-2.287; p<0.001, OR= 1.695, 95%CI= 1.335-2.152, respectively). For rs7923837, compared with individuals with AA, AG, GG, and AG/GG individuals had a significant increased CRC risk (p< 0.001, OR= 1.520, 95%CI= 1.200-1.924; p=0.036, OR= 1.739, 95%CI= 0.989-3.058; p< 0.001, OR= 1.540, 95%CI= 1.225-1.936, respectively). Significant difference in allele frequency distribution was observed between the control and CRC-only (p< 0.001, p=0.018). These results suggested that the rs1111875 and rs7923837 variant of HEEX gene were the risk factor of CRC, and it was independent on the occurrence of T2DM.

### Stratification analyses of rs1111875, rs7923837 and risk of CRC

The effects of rs1111875 or rs7923837 on CRC occurrence were further stratified by age, gender, smoking, drinking status and BMI. As shown in Table 5, the association between HHEX rs1111875 variant and CRC risk appeared stronger in subgroups of age< 55 (p< 0.001, OR=1.931, 95%CI =1.375-2.713), non-smokers (p< 0.001, OR=1.979, 95%CI =1.416-2.767), drinkers (P<0.001, OR=2.351, 95%CI =1.671-3.309), and BMI< 24 (P<0.001, OR=1.875, 95%CI =1.407-2.497). In both male and female subgroup, the association was significant (p= 0.022, OR=1.389, 95%CI=1.020-1.891; p< 0.001, OR=2.303, 95%CI=1.574 −3.369, respectively). As shown in Table 6, in both male and female, age≥55 and <55; never or ever smoking, drinking and BMI≥ 24 and <24 subgroup, the associations were significant.

**Table 5:** Stratification analyses of rs1111875 and risk of CRC

	TT		TC/CC		P*	Adjusted OR (95% CI)^#^
	Control n (304)	CRC-only n (244)	Control n (233)	CRC-only n (317)		
Gender						
male	166	162	138	187	**0.022**	1.389 (1.020-1.891)
female	138	82	95	130	**<0.001**	2.303 (1.574-3.369)
Age						
≥55	104	142	101	187	0.053	1.356 (0.955-1.925)
<55	200	102	132	130	**<0.001**	1.931 (1.375-2.713)
Smoking						
Never	155	121	121	134	**0.027**	1.419 (1.008-1.997)
Ever	149	123	112	183	**<0.001**	1.979 (1.416-2.767)
Drinking						
Never	139	121	136	147	0.120	1.242 (0.886-1.740)
Ever	165	123	97	170	**<0.001**	2.351 (1.671-3.309)
BMI						
≥24	101	76	77	75	0.147	1.294 (0.837-2.001)
<24	203	168	156	242	**<0.001**	1.875 (1.407-2.497)

* Two-sided χ2-test for the distributions of either genotype between the control and CRC-only crowd. ^#^Adjusted for age, gender, smoking status, drinking status in logistic regression model.

**Table 6:** Stratification analyses of rs7923837 and risk of CRC

	AA		AG/GG		P*	Adjusted OR (95% CI)^#^
	Control n (349)	CRC-only n (325)	Control n (221)	CRC-only n (317)		
Gender						
male	176	219	143	177	0.516	0.994 (0.740-1.338)
female	173	106	78	140	4.610	2.929 (2.028-4.231)
Age						
≥55	201	173	100	145	**0.001**	1.685 (1.216-2.334)
<55	148	152	121	172	**0.030**	1.384 (1.001-1.915)
Smoking						
Never	187	151	118	149	**0.004**	1.564 (1.132-2.160)
Ever	162	174	103	168	**0.007**	1.519 (1.097-2.103)
Drinking						
Never	209	163	114	138	**0.005**	1.552 (1.125-2.141)
Ever	140	162	107	169	**0.039**	1.365 (0.980-1.901)
BMI						
≥24	140	87	85	89	**0.007**	1.685 (1.130-2.514)
<24	209	238	136	228	**0.004**	1.472 (1.110-1.952)

* Two-sided χ2-test for the distributions of either genotype between the control and CRC-only crowd. ^#^Adjusted for age, gender, smoking status, drinking status in logistic regression model.

### Association between the rs1111875, rs7923837 polymorphism and the clinicopathological characteristics of CRC patients

The association between the rs1111875, rs7923837 polymorphisms and the clinicopathological parameters of CRC patients was presented in Table 7. The differences in rs1111875 genotype distribution were statistically significant in stagesII *vs.* IV (p=0.040, OR=1.792, 95%CI= 0.974-3.296), and in stages III *vs.* IV (p= 0.026, OR= 1.869, 95%CI=1.024-3.409). Similarly, the differences in rs7923837 genotype distributions achieved statistical significance among all stages (p = 0.043), and in stages II *vs.* III subgroups (p=0.004, OR= 1.628, 95%CI= 1.143-2.318). Besides, we also analyzed the association between the rs1111875, rs7923837 polymorphisms and the clinicopathological parameters of T2DM/CRC patients (Table S4). It is possible for the small T2DM/CRC sample size, no significant association was observed between different polymorphisms in either rs1111875 or rs7923837.

**Table 7:** The association of rs1111875, rs7923837 polymorphisms and clinical stage of CRC patients

Category			rs1111875				rs7923837	
Clinical stage	TT (244)	TC/CC (317)	p	OR (95% CI)	AA (325)	GA/GG (317)	p	Adjusted OR (95% CI)
I	27	37	0.212	1.00(reference)	31	37	**0.043**	1.00(reference)
II	93	112	0.382 (*vs.* I)	0.879 (0.498-1.550) (*vs.* I)	138	101	0.051 (*vs.* I)	0.613 (0.356-1.054) (*vs.* I)
III	110	127	0.323 (*vs.* I) 0.450 (*vs.* II)	0.842 (0.482-1.472) (*vs.* I) 0.959 (0.659-1.395) (*vs.* II)	120	143	0.553 (*vs.* I) **0.004 (*vs.* II)**	0.998 (0.584-1.705) (*vs.* I) 1.628 (1.143-2.318) (*vs.* II)
IV	19	41	0.152 (*vs.* I) **0.040 (*vs*. II)** **0.026 (*vs*. III)**	1.575 (0.754-3.288) (*vs.* I) 1.792 (0.974-3.296) (*vs.* II) 1.869 (1.024-3.409) (*vs.* III)	36	36	0.362 (*vs.* I) 0.153 (*vs.* II) 0.299 (*vs.* III)	0.838 (0.431-1.628) (*vs.* I) 1.366 (0.806-2.318) (*vs.* II) 0.839 (0.498-1.414) (*vs.* III)

* Two-sided χ2-test for the distributions of either genotype between each stage.

## DISCUSSION

The present study investigated the associations between 2 SNPs in HHEX and CRC susceptibility in a Chinese population. We found that that in T2DM patients, rs1111875 but not the rs7923837 variants in HHEX gene could be CRC risk factor. Additionally, our results also indicated that the two variants of HEEX gene could be risk factors for CRC in general population, independent of T2DM.

The mechanisms of increased risk for CRC in T2DM patients have been under extensive study. Both non-hereditary and hereditary factors have been elucidated. Among the non-hereditary mechanism are adipokines [16, 18], mitogenic effect of insulin [17, 19], the tumor-promoting effect of hyperglycemia and hyperinsulinemia in DM status, multiple usage of medication, diabetes-associated comorbidities, tissue-specific inflammation. For hereditary factors, HEEX attracts increasing attention in recent years [50]. Previous GWAS study has indicated that T2DM related variants HHEX rs7923837 could increase cancer risk in patients with diabetes [51].

In this study, we found that the increased CRC risk in T2DM patients compared with non-T2DM patients. Under the background of T2DM, the patients with the polymorphisms of rs1111875 T>C are susceptible to CRC than the wide type, which may give a clue about the association of T2DM susceptible SNPs and CRC. Interestingly, apart from the mechanism relying on the basic T2DM, the patients suffer from the HHEX polymorphism of rs1111875 T>C and rs7923837A>G are more prone to CRC. Polymorphisms of rs1111875 and rs7923937 are CRC risk factors, which could increase CRC susceptibility independent on the occurrence of T2DM.

Furthermore, we observed that the association between HHEX rs1111875 and increased CRC risk was more prominent in age< 55, non-smokers, drinkers and BMI< 24 subgroup, suggesting that the interaction of age, smoking and drinking status and genetic variants may affect the occurrence of CRC together. Thus, the association of HHEX rs1111875 and CRC occurrence was similar in both male and female subgroup, suggesting that the promotion effect of rs1111875 T>C polymorphism on CRC was not influenced by the gender. The association of HHEX rs7923837 variants and CRC occurrence was similar in both male and female, age≥55 and <55; never or ever smoking, drinking and BMI≥ 24 and <24 subgroup, suggesting that the effect of rs1111875 T>C polymorphism on CRC was not affect by the upper factors.

The expression of *HHEX* has been reported to be a fundamental signal for cell growth and development [27]. In line with this, we find significant association between HHEX rs7923837 variants and CRC staging. For rs11111875, the association was significant only between stage II and IV; stage III and IV, we speculate that the influence of rs1111875 is relatively subtle that may slowly promote the development of CRC, but not powerful enough to influence the CRC progression during the early stage of disease.

Since our case-control study was hospital based, we couldn't rule out a possibility of selection bias of these subjects who might be associated with a particular genotype. One of the strengths of this study is that the two-population based association studies between the Polymorphisms of rs1111875 T>C, rs7923837 A>G in HHEX polymorphisms and CRC risk provided sufficient statistical significance and reduce the false-positive report probability. However, several limitations should be addressed. Firstly, the subgroup analysis dealing with interactions between the HHEX genotype are based on the small number of subjects. As studies focused on Chinese are currently limited, further studies on these SNPs including a wider spectrum of subjects should be carried to investigate the role of these variants in different populations by larger prospective studies. Secondly, further functional studies of these two SNPs should be conducted to make a plausible biological explanation for the epidemiologic findings.

In summary, to our knowledge, this study provided the first evidence that the HHEX rs111875 T>C and rs7923837 A>G may contribute to an increased CRC risk in Chinese populations. However, our findings need to be validated by additional population-based prospective studies with different ethnic groups and well-designed clinical investigations. For future association studies, strict selection of patients, much larger sample size will be required. More studies should also be carried out to examine the impact of HHEX on CRC risk, especially in different populations.

## MATERIALS AND METHODS

### Ethics statement

The information obtained during the study did not affect the patients' diagnosis or treatment. The protocol was approved by the Committee on Research Ethics from PuAi Hospital of Tongji Medical College, Huazhong University of Science and Technology, and all subjects signed an informed consent term. Clinical investigation was conducted according to the principles expressed in the Declaration of Helsinki.

### Study population

In this study, 300 T2DM patients without secondary colorectal cancer (T2DM-only), 135 T2DM with secondary colorectal cancer patients (T2DM/CRC), 570 cancer-free controls (Control) and 642 colorectal cancer (CRC-only) cases were enrolled. All the cancer cases were histopathologically confirmed to be colorectal cancer for the first time and without history of other cancer and anti-cancer therapy. The disease was classified according to World Health Organization (WHO) criteria and were staged according to the American Joint Committee on Cancer (AJCC) TNM (tumor–node–metastasis) classification. Diabetes was characterized by persistent hyperglycaemia and was diagnosed by criteria defined in the ‘Diagnosis and classification of diabetes mellitus’ (American Diabetes Association) [43].

### Clinical and anthropometric profiles and laboratory analyses

A standard questionnaire was used to collect information from all patients about age, age at T2DM diagnosis, treatment and et al. All selected patients or volunteers underwent physical and laboratory evaluations. They were weighed (barefoot and wearing light outdoor clothing) and had their height measured. Body mass index (BMI) was calculated as body weight (kg) divided by square of height (m^2^). Whole blood samples were collected of all the health volunteer control or at the first visit of the T2DM patients, or before the first cycle of chemotherapy of the CRC patients. The levels of fasting serum glucose, insulin and HbA1c were assessed in the Department of Clinical Laboratory of our hospital. Insulin levels were measured by electro chemiluminescence immunoassay method using ADVIA Centaur XP Immunoassay System. Glucose levels were measured by GOD-POD assay using ADVIA 2400 Chemistry System. HbA1c was measured by high-performance liquid chromatography (HPLC) by a BIO-RAD D-10™ autoanalyzer according to the manufacture's instruction.

### Molecular analysis

DNA was extracted from peripheral blood leukocytes by a standardized salting-out procedure. Double-stranded direct sequencing was used to detect the selected SNPs of all the samples. Direct sequencing in an automated ABI 3100 Avant Genetic Analyzer (Life Technologies, Foster City, CA, USA) was performed using ABI Prism Big Dye Terminator Cycle Sequence Ready reaction kit (Life Technologies) according to the manufacturers' recommendations, and using primers described in Table S3. The results generated by sequencing were compared with the sequences of the human gene sequence available in GenBank (http://www.ncbi.nlm.nih.gov/genbank/). The representative sequencing results of the three genotypes of rs1111875 and rs7923837 were described in Figure S1 and Figure S2.

### Statistical analyses

Statistical analysis was performed with SPSS version 13.0 (SPSS Institute, Chicago, IL). The χ^2^ test was used to compare categorical data including gender, SNP cases, et al. Two sided P < 0.05 was considered as statistically significant. Allelic frequencies were determined by gene counting, and departures from the Hardy-Weinberg equilibrium (HWE) were investigated using chi-squared test and P < 0.05 indicated deviation from the equilibrium.

## SUPPLEMENTAL TABLES AND FIGURES



## Abbreviations

CRC: colorectal cancer
DM: Diabetes Mellitus
T2DM: Type 2 Diabetes Mellitus
HHEX: hematopoietically-expressed homeobox
T2DM-only: T2DM patients without secondary colorectal cancer
T2DM/CRC: T2DM patients with secondary colorectal cancer
CRC-only: CRC patients without the DM history.
