# Supplementary material for: Two variants on T2DM susceptible gene HHEX are associated with CRC risk in a Chinese population
Source: Oncotarget. 2016 Apr 20;7(20):29770–9. doi: 10.18632/oncotarget.8865 (PMC5045432; doi:10.18632/oncotarget.8865)
Supplement: Supplementary file 1 [file oncotarget-07-29770-s001.pdf]

Two variants on T2DM susceptible gene HHEX are associated with CRC risk in a Chinese population

SUPPLEMENTAL TABLES AND FIGURES

Supplementary Table S1: Frequency distribution of selected variables between the T2DM/CRC cases and T2DM-only subjects

Variables	T2DM-only (n=300)		T2DM/CRC (n=135)		p
	N	%	N	%	
Age (year)	58.0±12.6		57.5±13.4		0.71
BMI	24.4±3.2		23.8±2.8		0.06
FSG (mmol/L)	8.4±3.6		8.6±4.1		0.61
Insulin (pmol/L)	13.2±6.6		12.1±5.8		0.10
HbA1c (%)	6.7±1.8		6.5±1.6		0.27
Gender					
Male	145	48.3	55	40.7	0.14
Female	155	51.7	80	59.3	
Smoking					
Never	172	57.3	75	55.6	0.73
Ever	128	42.7	60	44.4	
Drinking					
Never	142	47.3	45	33.3	0.006
Ever	158	52.7	90	66.7	
HBP					
No	204	68.0	99	73.3	0.26
Yes	96	32.0	36	26.7	
Clinical stage					
I			33	24.5	
II			20	14.8	
III			50	37.0	
IV			32	23.7	

Supplementary Table S2: Frequency distribution of selected variables between the control and CRC-only subjects

Variables	control (n=570)		CRC-only (n=642)		p
	N	%	N	%	
Age (year)	58.6±12.6		57.4±11.8		0.08
BMI	24.8±3.2		24.6±3.4		0.29
FSG (mmol/L)	5.2±1.5		5.1±1.8		0.30
Insulin (pmol/L)	76.2±33.4		73.5±38.5		0.20
HbA1c (%)	4.6±1.1		4.5±1.2		0.13
Gender					
Male	349	61.2	362	56.4	0.09
Female	221	38.8	280	43.6	
Smoking					
Never	302	53.0	302	47.0	0.04
Ever	268	47.0	340	53.0	
Drinking					
Never	320	56.1	337	52.5	0.09
Ever	250	43.9	305	47.5	
HBP					
No	399	70.0	421	65.6	0.09
Yes	171	30.0	221	34.4	
Clinical stage					
I			158	24.6	
II			88	13.7	
III			220	34.3	
IV			176	27.4	

Supplementary Table S3: The sequence of the primers used in the PCR and sequencing experiments

SNPs		primers	nt	product length
rs1111875	sense:	5'-TGAAATAACATCTAAACAAGGGGCAGCT-3'	28	317
	antisense:	5'-AGTTTCAACTTTCCTTAGCTGCTTATGG-3'	28	
rs7923837	sense:	5'-CAATAAGAGAAGGCACACATAGTGCTTAG-3'	29	288
	antisense:	5'-CCAATACCTTGGCCTGGCTCTTG-3'	23	

Supplementary Table S4: The association of rs1111875, rs7923837 polymorphisms and clinical stage of T2DM/CRC patients

Category	rs1111875				rs7923837			
Clinical stage	TT (60)	TC/CC (69)	p	OR (95% CI)	AA (87)	GA/GG (48)	p	Adjusted OR (95% CI)
I	7	8	0.689	1.00(reference)	8	6	0.349	1.00(reference)
II	22	24	0.823 (vs I)	0.954 (0.297-3.069) (vs I)	37	13	0.222 (vs I)	0.468 (0.136-1.607) (vs I)
III	27	28	0.887 (vs I)<br>0.920 (vs II)	0.907 (0.289-2.848) (vs I)<br>0.951 (0.434-2.081) (vs II)	32	23	0.823 (vs I)<br>0.134 (vs II)	0.958 (0.293-3.139) (vs I)<br>2.046 (0.893-4.684) (vs II)
IV	4	9	0.639 (vs I)<br>0.438 (vs II)<br>0.377 (vs III)	1.969 (0.416-9.317) (vs I)<br>2.063 (0.555-7.661) (vs II)<br>2.169 (0.597-7.890) (vs III)	10	6	0.920(vs I)<br>0.377 (vs II)<br>1.000 (vs III)	0.800 (0.185-3.461) (vs I)<br>1.708 (0.518-5.631) (vs II)<br>0.835 (0.266-2.624) (vs III)

Two-sided χ2-test for the distributions of either genotype between each stage.

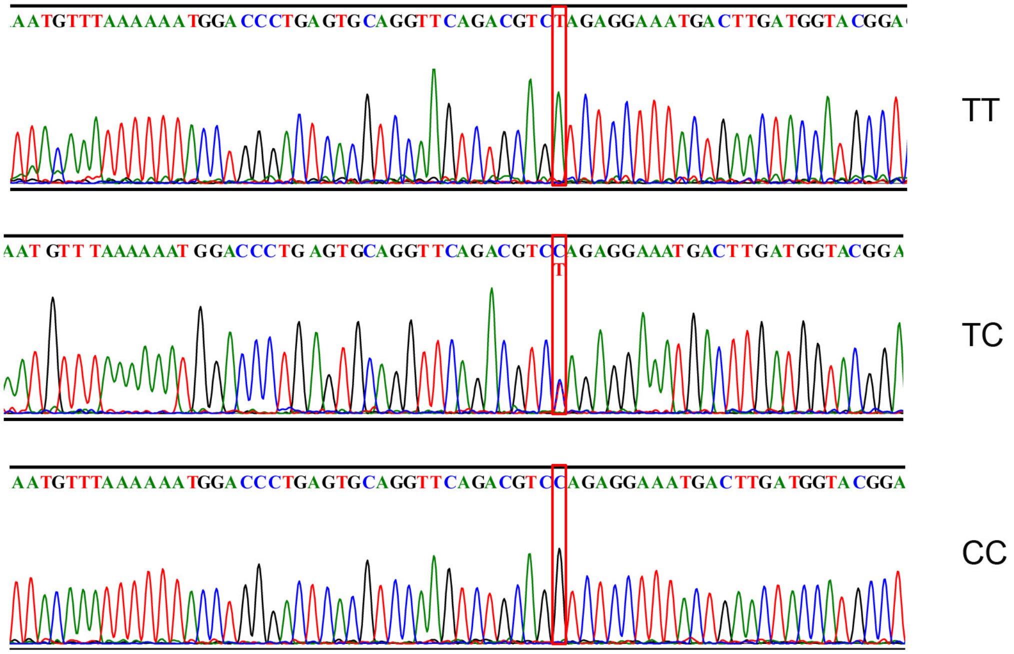

Supplementary Figure S1: The representative sequencing results of TT, TC, CC genotype of rs1111875. Red box: the rs1111875 allele site.

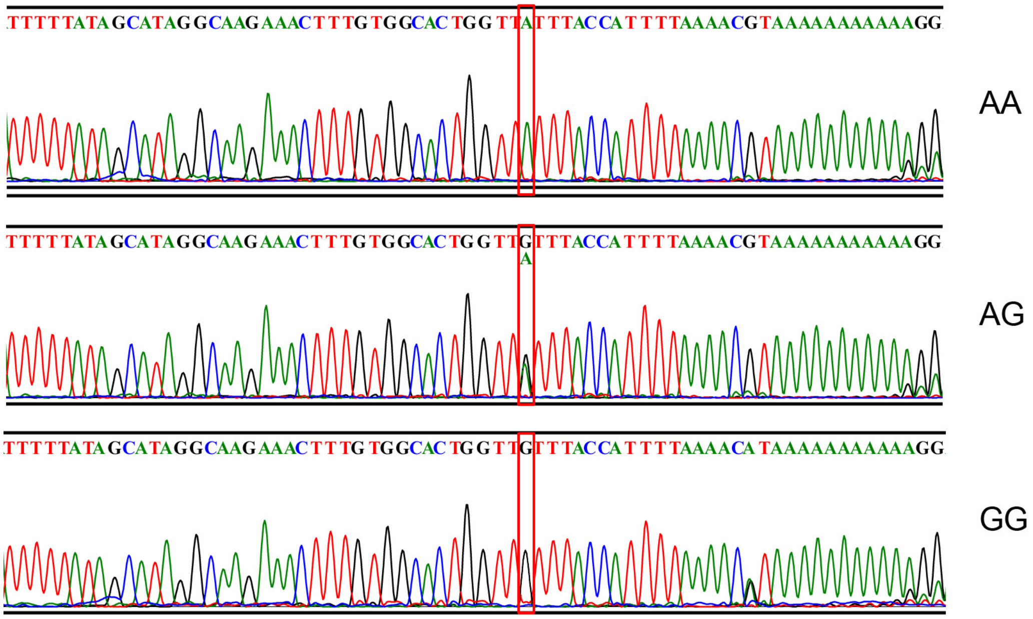

Supplementary Figure S2: The representative sequencing results of AA, AG, GG genotype of rs7923837. Red box: the rs7923837 allele site.
